# A new method of continuous blood pressure monitoring using multichannel sensing signals on the wrist

**DOI:** 10.1038/s41378-023-00590-4

**Published:** 2023-09-21

**Authors:** Liangqi Wang, Shuo Tian, Rong Zhu

**Affiliations:** State Key Laboratory of Precision Measurement Technology and Instrument, Department of Precision Instrument, Tsinghua University, 100084 Beijing, China

**Keywords:** Electrical and electronic engineering, Materials science

## Abstract

Hypertension is a worldwide health problem and a primary risk factor for cardiovascular disease. Continuous monitoring of blood pressure has important clinical value for the early diagnosis and prevention of cardiovascular disease. However, existing technologies for wearable continuous blood pressure monitoring are usually inaccurate, rely on subject-specific calibration and have poor generalization across individuals, which limit their practical applications. Here, we report a new blood pressure measurement method and develop an associated wearable device to implement continuous blood pressure monitoring for new subjects. The wearable device detects cardiac output and pulse waveform features through dual photoplethysmography (PPG) sensors worn on the palmar and dorsal sides of the wrist, incorporating custom-made interface sensors to detect the wearing contact pressure and skin temperature. The detected multichannel signals are fused using a machine-learning algorithm to estimate continuous blood pressure in real time. This dual PPG sensing method effectively eliminates the personal differences in PPG signals caused by different people and different wearing conditions. The proposed wearable device enables continuous blood pressure monitoring with good generalizability across individuals and demonstrates promising potential in personal health care applications.

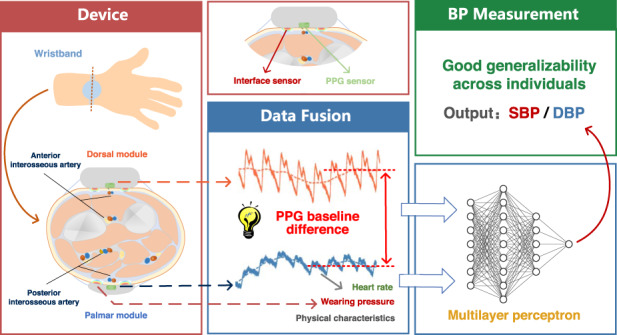

## Introduction

Hypertension is a serious disease that greatly increases the risk of cardiovascular disease and is an important cause of premature death worldwide. The number of people aged 30–79 years with hypertension has reached 1.28 billion, and about half of these people do not know they have hypertension^1^. The timely detection of blood pressure (BP) and the prevention of cardiovascular disease are therefore of great significance to human health. Importantly, wearable devices used for continuous blood pressure monitoring in daily home care are imperative and have a huge market demand.

Blood pressure is the pressure of circulating blood against the walls of blood vessels. The bulk of our knowledge about blood pressure is based on traditional recording methods of taking a small number of readings with manual auscultation or oscillometric techniques (in a medical setting or at home)^2^. However, such measurements, which are of enormous value on a population basis, often provide a poor estimate of blood pressure in a person, and are limited by factors such as poor technique of the observer, the “white coat” effect (i.e., the transient but variable evaluation of blood pressure in a medical setting)^3^, and the inherent variability of blood pressure^4^.

Ambulatory blood pressure monitoring has thus been developed to improve the monitoring of true blood pressure^5,6^. The available ambulatory blood pressure monitors use the oscillometric technique. These bulk monitors are typically worn on a belt or in a pouch and are connected to a cuff on the upper arm by a plastic tube. The monitors take readings every quarter to half an hour. Ambulatory blood pressure monitoring can estimate true or mean blood pressure, the diurnal rhythm of blood pressure, and blood pressure variability. However, it cannot provide continuous blood pressure measurements. Without continuous monitoring, the dynamic BP response to daily physical and mental activities is unknown.

Continuous blood pressure measurements have attracted public attention and been widely investigated^5^. Volume clamping and tonometry are two conventional techniques for continuous blood pressure monitoring. However, they require constant pressure on the skin, and long-term use may cause discomfort. For example, volume clamping requires the device to be worn on the finger, causing inconvenience. Moreover, tonometry is sensitive to motion artifacts and requires frequent calibrations.

To solve the above problems of existing continuous blood pressure measurements, some methods have been proposed, including pulse transit time (PTT)^7,8^, pulse arrival time (PAT)^9^, pulse wave velocity (PWV)^10^, pulse wave analysis (PWA)^11^, facial video method^12,13^ and ultrasound method^14,15^. The PWV approach is based on the arrival time of a pressure wave propagating through the arterial tree at a certain distance between the proximal and distal arterial sites in the form of PWV = L/PTT, where L is the distance between the proximal and distal sites. When blood pressure increases, the vascular tone increases, and the arterial wall becomes stiffer, causing the PTT to shorten^5^. Conversely, when blood pressure falls, vascular tone decreases, the arterial wall becomes less stiff, and PTT increases. By using a calibration procedure, the measured PTT can be translated into arterial pressure by using an appropriate model. The proximal waveform can be measured by photoplethysmography (PPG)^16^, pressure sensors, cardiography (ICG)^17^, ballistocardiography (BCG)^18^, seismocardiography (SCG)^19^, etc. The method is called PAT when electrocardiography (ECG) is used to obtain a surrogate of the proximal waveform. PWA involves extracting features from an arterial waveform and mapping them to BP units via a calibration model (Ramakrishna et al. 2022). PWA is more convenient than the PTT method since only a single sensor is needed, and it can be combined with PTT to improve its accuracy. Benefiting from the popularity of machine learning, the PWA method has garnered increasing attention. However, PTT, PAT, PWV, and PWA are data-driven methods, have poor generalization across different individuals, and need frequent subject-specific calibrations. The facial video method uses facial video to extract arterial waveform features and uses PWA or PTT to estimate blood pressure. The ultrasound method^14^ records the diameter of a pulsating blood vessel, but it requires calibration with a cuff sphygmomanometer.

Despite the existence of the off-the-shelf methods of continuous blood pressure measurements mentioned above, existing approaches face a common bottleneck problem of poor generalization across different individuals and different wears. Blood pressure measurements are often evaluated after subject-specific calibration. Few wearable methods can achieve the AAMI standards (mean error (ME) < ± 5 mmHg and standard deviation (SD) < 8 mmHg) across a generalized population of continuous blood pressure measurement for new subjects. However, practical healthcare application requires blood pressure monitoring without relying on subject-specific calibration, which is necessary for large-scale deployment.

Here, we propose a new BP measurement method and develop a bilateral wristwatch device composed of dual PPG sensors and custom-made interface sensors. The proposed device enables noninvasive continuous blood pressure measurement with good generalizability across new subjects and different wears without relying on subject-specific calibration. The wearable device utilizes dual PPG sensors worn on the palmar and dorsal sides of the wrist to detect the cardiac output and the pulse waveform features and interface sensors to detect the wearing pressure and skin temperature. The detected multichannel signals are fused using a machine-learning algorithm to estimate continuous BP in real time for wearers. Dual PPG sensing on the wrist can eliminate the personal differences in PPG signals caused by different people and different wearing conditions and thus enhance the generalizability of the BP measurement across individuals. The simultaneously detected contact pressure and skin temperature further enhance the generalizability of the BP measurement.

## Materials and methods

### Measurement principle of continuous blood pressure

**Fig. 1:**
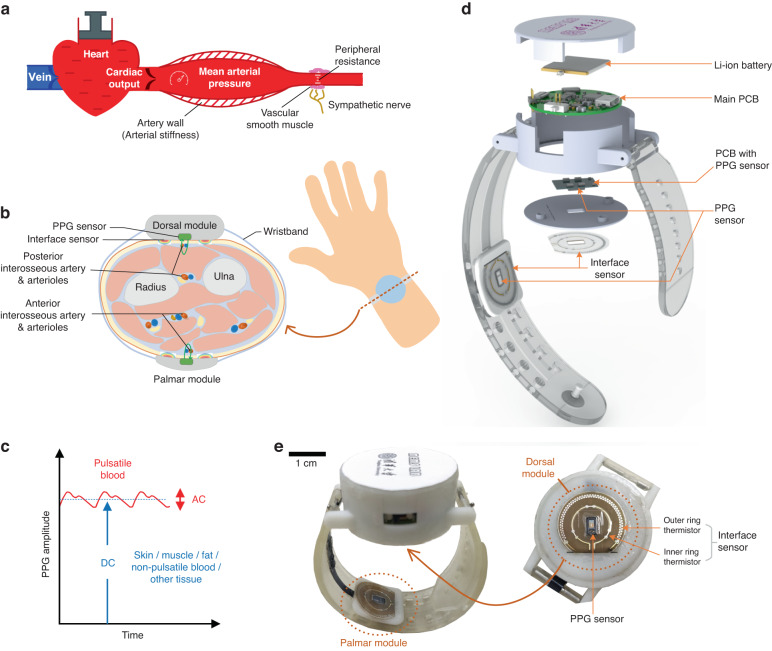
Measurement principle and wearable device. **a** Mechanism of arterial blood pressure. Blood pressure is mainly determined by cardiac output and systemic vascular resistance. **b** Schematic diagram of blood pressure measurement by the proposed method. **c** The DC and AC components of the PPG signal. **d** The exploded view of the wearable device. **e** Prototype of the wearable device, including the overall device and the bottom surface of the device

Mean blood pressure is mainly determined by cardiac output and systemic vascular resistance^20^. Pulse pressure depends mainly on the viscoelastic properties of large elastic arteries, which represents the fluctuation in pressure values around the mean blood pressure^21^ (Fig. 1a). Systolic blood pressure (SBP) is the blood pressure when the heart pumps. Diastolic blood pressure (DBP) is the blood pressure when the heart is resting. Cardiac output is the volumetric flow rate of the heart’s pumping output, which is the product of the heart rate, and the stroke volume, which is the volume of blood pumped from the left ventricle per beat. When other factors remain unchanged, as the stroke volume increases, the volume of blood injected into the aorta during systole increases, and the pressure on the artery wall increases, resulting in an increase of SBP. Due to the increase of arterial blood pressure, the blood flow speed is accelerated, and the amount of blood left in the artery at the end of the diastole does not increase much, resulting in a little change of DBP. In contrast, when the stroke volume decreases, the SBP decreases significantly, but the DBP decreases slightly. When the heart rate increases, the SBP increases slightly, and the DBP increases significantly. Systemic vascular resistance mainly affects DBP. The greater the peripheral resistance is, the higher the DBP. The elasticity of the aortic wall buffers the change of blood pressure. As it decreases, the SBP increases and the DBP decreases.

Our proposed device detects cardiac output using dual PPG sensors worn on the palmar and dorsal sides of the wrist (Fig. 1b). In circulation, the volume of blood changes periodically as the heart beats. This volume reflects important cardiovascular information, such as cardiac function, blood flow, peripheral blood, microcirculation, etc., and is called volume pulse wave. It is usually detected using a PPG sensor. As shown in Fig. 1c, the variable component of the PPG signal is designated AC, which is induced by pulsatile blood. The DC signal is induced by the static absorption of skin, tissues, nonpulsatile blood, etc. The DC component is larger than the AC component. Since the human cardiovascular system is a closed system, the overall integrated value of volumetric pulse flow during each cardiac cycle is equal to the single-cycle cardiac output, i.e., the volume per beat. The DC component of volumetric pulse flow largely reflects the magnitude of the volume per beat, a measure of blood flow parameters such as vascular resistance and vascular elasticity. Although the AC component accounts for a small part of the PPG signal, it can reflect changes in cardiac ejection, peripheral resistance, and so on^22,23^. This indicates that the estimate of SBP is correlated with the DC component of the PPG signal through the correlation of volume per beat, while the estimate of DBP is correlated with the AC and DC components of the PPG signal through the correlation of pulse waveform and peripheral resistance. However, this correlation does not represent an absolute linear relationship between the two. In terms of physiological mechanisms, stroke volume is one of the factors that affects the DC components of PPG signals and SBP, and vascular parameter such as peripheral resistance is one of the factors that affects the AC/DC components of PPG signals and DBP. However, there are also confounding factors, such as contact pressure and skin color, that affect PPG signals, which make the estimation of SBP/DBP from the AC/DC components of PPGs less accurate. Therefore, contact pressure and skin temperature need to be incorporated into the compensation when performing blood pressure estimation.

Due to individual differences in skin colors, tissues, muscles, and other factors, the amount of light emitted by the light-emitting diode (LED) of the PPG sensor varies greatly across subjects. Moreover, even if the same person wears the PPG sensor, the PPG signal is also different across different wears due to uncertain tightness and drifts with skin temperature. For these reasons, it is difficult to extract the DC component of the PPG signal relevant to the cardiac output by a single PPG sensor. To solve this problem, we propose a new method to make a difference calculation of DC components of dual PPG signals detected simultaneously on the palmar and dorsal sides of the wrist to extract the SBP related to the cardiac output.

The PPG sensor on the palmar side of the wrist measures the arterioles near the anterior interosseous artery, and the PPG sensor on the dorsal side of the wrist measures the arterioles near the posterior interosseous artery. Both arteries are branches of the common interosseous artery, such that many communications exist between them, and these communications can be observed by means of a periosteal network on the palmar aspect of the forearm^24^. This correlation, together with the fact that the distances from the measurement locations on both arteries to the branch of the brachial artery are essentially the same, ensures the temporal synchronization of blood flow changes at both measurement locations. Therefore, the stroke volume can be reflected in the difference of blood flows between the two vessels. Since the two PPG sensors are worn on the same wrist, the skin color, tissue, and muscle have similar effects on light absorption. The differential operation can reduce the effect of these personal features. Therefore, the DC difference of dual PPG sensor signals can reflect the stroke volume information, and differential processing can also eliminate the offset influence of hydrostatic pressure caused by gravity. This dual PPG method can reduce the personal differences in PPG signals caused by different people and different wearing conditions. In addition to the DC component of the PPG signal, the AC component can also be used for pulse waveform analysis. Physical characteristics such as the subject’s heart rate, weight, age, height, sex, and body mass index also help to estimate blood pressure. Another important aspect of device performance is the wearing condition. When the PPG sensor is worn on the wrist, the light absorption ratio of the skin, muscle, and blood vessel changes with the contact pressure^25–28^, which affects the baseline and waveform of the PPG signal. The contact pressure between the PPG sensor and the skin can be detected using our custom-made thermosensation-based interface sensor^29^. Meanwhile, our interface sensor can also measure skin temperature. A negative correlation has been reported between blood pressure and skin temperature^30,31^.

Based on the above analysis, we utilize the DC difference of dual PPG signals at the wrist, contact pressure signals detected by the interface sensor, and subjects’ physical characteristics to estimate SBP. We further utilize the DC difference and waveform features of dual PPG signals at the wrist, contact pressure signals, and subjects’ physical characteristics to estimate DBP. Therein, our estimation method uses data fusion based on machine learning.

### Device design

**Fig. 2:**
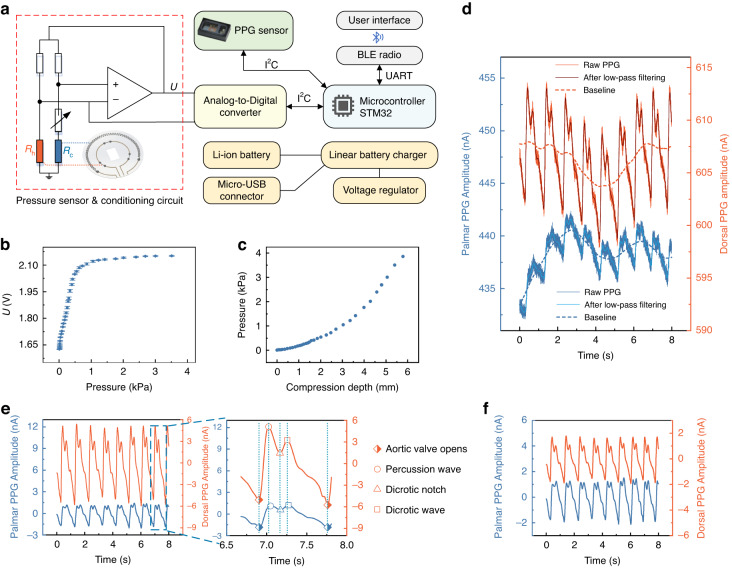
System structure, pressure sensing characterizations, and PPG signal processing. **a** Block diagram of the electronic system of the wearable device. The module outlined in red dashes is the CTD circuit of the interface sensor. **b** Contact pressure sensing response of the interface sensor. The error of the contact pressure measurement is less than 18 Pa. **c** Measured contact pressure under different compression depths. **d** Raw PPG signals from the palmar and dorsal sensors, signals after low-pass filtering and wavelet denoising, and the baselines. **e** The AC components of PPG signals and comparison of each characteristic peak moment of the pulse. **f** PPG signals after z-score standardization

The device is designed similar to a wristwatch, as shown in Fig. 1b, d, e. The device case is made of resin and 3D printed (3.8 cm diameter, 1.3 cm thickness). Two sets of PPG sensors and interface sensors are mounted on the back surface of the wristwatch device and the wristband, respectively, which are utilized to measure the arterial pulse PPG signals and wear pressures on opposite sides of the wrist. The PPG sensor is a reflectance type (MAX30101, Maxim) with a photodetector and a 525 nm (green) LED. The interface sensor is coplanar with the PPG sensor. The circuit of the sensors is composed of an analog circuit and a digital circuit, and its schematic diagram is shown in Fig. 2a. The analog circuit is mainly used to amplify and filter the interface sensor signals. Output signals of all sensors are collected by a microcontroller unit (MCU: STM32L452, STMicroelectronics) via an analog-to-digital converter (ADS124S06, Texas Instruments). A Bluetooth low energy module (DA14580, Dialog Semiconductor) is used for data communication between the device and a PC. The sampling rates of the PPG sensors and interface sensors were 1 kHz and 125 Hz, respectively. The wearable device has a power consumption of 290 mW. A rechargeable lithium-ion battery is used for the power supply. The device can work continuously for more than 4 h using a 300 mAh lithium-ion battery. The circuit has a battery charger IC, and the battery can be charged through the Micro-B USB connector plug.

The interface sensor utilizes a thin-film thermistor to generate a thermal field and maintain a constant temperature using a constant temperature difference (CTD) feedback circuit (Fig. 2a). The sensor is flexible and thin (50 μm) and composed of two concentric circular ribbons (i.e., thin-film thermistors made of platinum) deposited on a flexible polyimide substrate (the detailed fabrication process is shown in Supplementary Note S1). The ribbon of the inner ring (total 40 Ω, three Pt thermistors connected in series by copper wires) is electrically heated to a predefined higher temperature (5 K) than the surrounding and works as a contact pressure sensor. Meanwhile, the ribbon of the outer ring (2000 Ω, a Pt thermistor) functions as a temperature sensor. The details of the resistances of the sensor are shown in Supplementary Fig. S1.

When the interface sensor contacts human skin, there is a conductive heat transfer from the heated ribbon of the inner ring to the skin. Conductive heat transfer is positively correlated with the thermal conductivity of the skin. Take advantage of the skin’s natural piezo-thermic transduction, i.e., the contact pressure can be converted to a change in the skin’s thermal conductivity and detected by the thermistor of the inner ring. Specifically, the contact pressure compresses the skin and changes its thermal conductivity, and thus, the heat conduction between the interface sensor and the skin changes. Therefore, the interface sensor responds to the contact pressure. Using this CTD scheme, contact pressure sensing is immune to temperature fluctuations. The principle of temperature compensation by the CTD scheme is described in Supplementary Note S2 and Supplementary Fig. S2, and the performance of temperature compensation for pressure sensing is shown in Supplementary Fig. S3.

To characterize the interface sensing capability, the contact pressure and compression depth sensing responses are measured (the detailed experimental process is described in Supplementary Note S3). Figure 2b illustrates the interface sensor response to the contact pressure. The results indicate that the output voltage of the interface sensor increases with the contact pressure. The pressure sensing range of 4 kPa covers the tightness of the wristwatch’s wear. Figure 2c demonstrates the relationship between the contact pressure and compression depth, which characterizes the equivalent Young’s modulus of human skin. The results indicate that the equivalent Young’s modulus of human skin increases with the compression depth. In addition to sense the contact pressure, the interface sensor also detects skin temperature by the thermistor of the outer ring, as shown in Supplementary Fig. S4. The relevant principle underlying our temperature measurement is described in Supplementary Note S4.

### Data fusion method for continuous blood pressure measurement

Continuous blood pressure measurement is conducted by wearing our device on the wrist of the subject, while the ground truth blood pressure is measured simultaneously using a commercial cuff-based blood pressure monitor, as shown in Supplementary Fig. S5. In the experiment, the dual PPG signals and interface sensor signals are collected in real time, and the SBP/DBP are estimated using a 10-s window with 2-s shifts (8-s overlap), yielding the SBP/DBP every 2 s. The raw PPG signals are shown in Fig. 2d, which contain high-frequency noises and baseline drifts. The high-frequency noise is mainly induced by the PPG sensor, and the baseline drift is caused by respiration, skin temperature variation, and other factors. The clear PPG signals and baselines are obtained by using low-pass filtering and wavelet denoising. After removing the baselines, the pulsatile components of the PPG signals are shown in Fig. 2e. A z-score standardization is further performed to transform their mean value to 0 and standard deviation to 1^32^. The waveforms are shown in Fig. 2f. In the subsequent waveform feature extraction, the PPG signals are downsampled to 500 Hz, and in the subsequent deep learning, the PPG signal is further downsampled to 50 Hz to reduce the computational load (the detailed process is shown in Supplementary Note S5). Figure 2d, e, f also illustrate the temporal synchrony of the dual PPG signals on the dorsal and palmar sides of the wrist, indicating the temporal synchronization of the blood flows at both measurement locations.

The classical PTT method usually requires the two PPG signals to be measured at locations far enough apart to obtain a more significant time difference to be used for blood pressure estimation. In our measurement, we utilize dual PPG signals on the dorsal and palmar sides of the wrist, and there is temporal synchrony between the two signals as observed from the point of aortic valve opening as the pacing point of a cycle. This is because the corresponding arteries at both measurement locations are branches of the common interosseous artery, and the communication between the two are observed using a periosteal network on the palmar aspect of the forearm. Because the distances to the branches of the brachial artery are essentially the same at both measurement locations, the changes in the blood flows at both measurement locations are temporally synchronous. For features such as the dicrotic notch and dicrotic wave, which contain more information about the reflected waves, there is a stable difference in the PPG signal between the two locations. In Fig. 2e, the PPG signal measured on the palmar side shows characteristic peaks that are later than the dorsal PPG signal. This, combined with the similar amplitudes of the percussion wave and dicrotic wave on the palmar side of the hand, indicates that the vascular resistance at the posterior end of the anterior interosseous artery is greater than that at the radial artery. There is a difference in the waveforms of the PPG signals on the two sides due to the difference in the reflected waves. In contrast, differential analysis based on the DC components of PPG signals can avoid the individualized differences in waveform analysis and parameter extraction introduced by the differences in posterior vascular resistance.

**Fig. 3:**
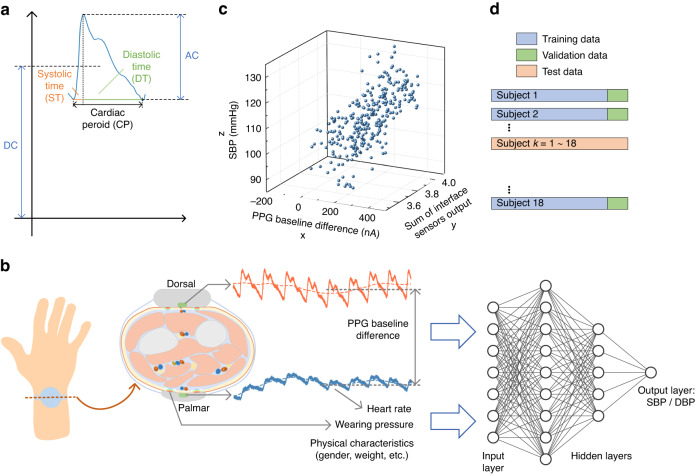
Feature extraction and data fusion. **a** Features extracted from the PPG signal. **b** Data fusion method: multilayer perceptron with 2 hidden layers. **c** PPG baseline difference and output sum of interface sensors have a positive correlation with SBP. The x-axis is the PPG baseline difference, the y-axis is the output sum of interface sensors, and the z-axis is the SBP. **d** Schematic view of the leave-one-subject-out (LOSO) cross-validation approach for validating generalizability across subjects

The PPG pulsatile component is characterized by its height and the duration of specific components of the cardiac cycle. However, pulse height is not a reliable clinical parameter, as it may vary significantly across different subjects due to personal factors during PPG signal recording^33^. Based on the aforementioned blood pressure measurement principle, 8 pulse features are extracted from the palmar and dorsal PPG signals, including a baseline difference of dual PPG signals, cardiac period (CP), systolic time (ST), diastolic time (DT), the ratio of ST and DT, the ratio of systolic area and diastolic area, K value, and the ratio of AC and DC components. Some of these pulse features are illustrated in Fig. 3a. These 8 features are defined from a theoretical basis (definition details underlying these features are provided in Supplementary Note S6). These pulse features are extracted from the dorsal PPG signal due to its higher signal-to-noise ratio. They are extracted from every cardiac period in the time window, and the mean value is calculated. The features of contact pressure and skin temperature are extracted from the signals of the palmar and dorsal interface sensors.

We combine 8 features of PPG signals, 4 features of interface sensor signals, and the subject’s physical characteristics (heart rate, age, height, weight, BMI index, gender), a total of 18 features, to make a data fusion for estimating continuous blood pressure.

Due to complex correlations between multiple signal features and blood pressure, a machine learning method is generally used to establish a correlation model between multiple features and blood pressure. Here, a neural network based on Keras framework is adopted to build the data fusion model, and the framework is based on TensorFlow. A multilayer perceptron (MLP) is used to estimate blood pressure, as shown in Fig. 3b. The extracted multiple features are the inputs of MLP, and the output is SBP or DBP. The hidden layer is set as 2 layers, and the numbers of neurons are optimized to be 80 and 12. Behind each hidden layer, there is a dropout layer (dropout layer 20%) to increase generalization ability and reduce overfitting. Rectified linear units (ReLU) are set as the activation function of the hidden layer, and the mean squared error (MSE) is the loss function. The optimizer is Adam algorithm, and the learning rate is 0.001. The optimization process for the neural network is described in Supplementary Note S7.

## Results and discussion

### Experiment of blood pressure measurement

To verify the effectiveness of the proposed blood pressure measurement method, a total of *N* = 18 healthy subjects (age: 24.8 ± 3.1 years, BMI: 21.1 ± 2.6 kg/m^2^) participate in the experiment (*N* = 13 male and *N* = 5 female). A total of 309 datasets of blood pressure measurements on 18 subjects are collected. The subjects remove and rewear the monitoring device between different measurements to validate the feasibility and generalization across different wears. The details of the blood pressure measurements are provided in Supplementary Note S8, and the blood pressure distribution is shown in Supplementary Fig. S6.

We seek to develop a generalized wearable continuous blood pressure measurement that is applicable to different individuals. Our method is validated based on the testing performance using the leave-one-subject-out cross-validation approach (LOSO). Specifically, each subject’s dataset is taken successively as the test set (new subject), 85% of the remaining 17 subjects’ datasets are taken as the training set and 15% as the validation set, as shown in Fig. 3d. The estimated values obtained are summarized for statistical assessments (ME and SD, defined in Supplementary Note S9). For comparison, the estimation of the SBP and DBP without LOSO are also provided, wherein 70% of the 18 subjects’ datasets are taken as the training set to establish a generalized model for 18 subjects and the remaining 15% are taken as the validation set, with the final 15% used for testing.

The input parameter configuration of the MLP model for estimating blood pressure is crucial and needs to be optimized. The input parameters are selected from the features of the dual PPG baseline difference, wearing contact pressure signals and skin temperature signals (palmar and dorsal), subject’s physical characteristics (heart rate, age, height, weight, BMI index, and gender), and PPG waveform features set (7 waveform features). After data cleansing, we observe that the baseline difference in the dual PPG signals and the detected wearing pressure are positively correlated with SBP, as shown in Fig. 3c. The total wearing pressure of the dorsal module and the palmar module is determined by summing the output voltages of the two interface sensors. The input parameter configuration for estimating blood pressure is optimized in Supplementary Note S10. Through the optimization, PPG baseline difference, contact pressure signals, skin temperature signals, and the subject’s physical characteristics are used to estimate SBP. The PPG baseline difference, contact pressure signals, skin temperature signals, PPG waveform features set, and the subject’s physical characteristics are used to estimate DBP.

### Results of blood pressure estimation

**Fig. 4:**
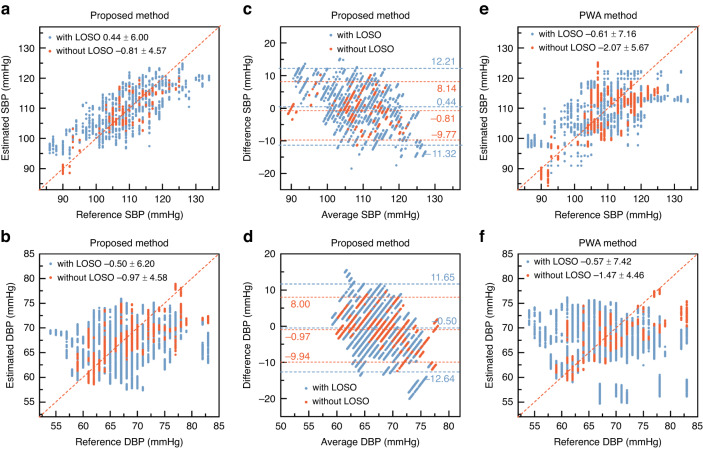
Scatter plots and Bland‒Altman plots of SBP and DBP estimates for 18 subjects with and without LOSO. **a** Scatter plot between the reference SBP and the estimated SBP using the proposed method with LOSO and without LOSO. **b** Scatter plot between the reference DBP and the estimated DBP using the proposed method with LOSO and without LOSO. **c** Bland‒Altman plot of the estimated SBP using the proposed method. With LOSO, the mean is 0.44 mmHg, the standard deviation is 6.00 mmHg and the LOA is [−11.32, 12.21] mmHg. Without LOSO, the mean is −0.97 mmHg, the standard deviation is 4.57 mmHg, and the LOA is [−9.77, 8.14] mmHg. **d** Bland‒Altman plot of the estimated DBP using the proposed method. With LOSO, the mean is −0.50 mmHg, the standard deviation is 6.20 mmHg, and the LOA is [−12.64, 11.65] mmHg. Without LOSO, the mean is −0.97 mmHg, the standard deviation is 4.58 mmHg, and the LOA is [−9.94, 8.00] mmHg. **e** Scatter plot between the reference SBP and the estimated SBP using the PWA method with LOSO and without LOSO. **f** Scatter plot between the reference DBP and the estimated DBP using the PWA method with LOSO and without LOSO

The MLP model for estimating SBP and DBP is trained and tested with LOSO, which means that the generalization ability of our method is evaluated. The experimental results indicate that the estimation errors are 0.44 ± 6.00 mmHg (ME ± SD) for SBP and −0.50 ± 6.20 mmHg (ME ± SD) for DBP, as shown in Fig. 4a, b with blue data points. In addition, a Bland‒Altman plot is also used to verify the agreement between the estimated blood pressure and the actual blood pressure, as shown in Fig. 4c, d with blue data points. The limit of agreement (LOA) is expressed by [μ − 1.96σ, μ + 1.96σ], where μ and σ are the mean and the standard deviation of the blood pressure difference between the estimated blood pressure and true blood pressure, respectively. The LOA is [−11.32, 12.21] mmHg for SBP and [−12.64, 11.65] mmHg for DBP. There are few outliers for both blood pressure estimates. The estimation errors of SBP and DBP achieve the standard of AAMI (ME < ± 5 mmHg and SD < 8 mmHg) and BHS Grade B. For comparison, the estimation results without LOSO are also shown as orange data points. The estimation errors are −0.81 ± 4.57 mmHg for SBP and −0.97 ± 4.58 mmHg for DBP, as shown in Fig. 4a, b with orange data points. In addition, the Bland‒Altman plots verify the agreement between the estimated blood pressure and the actual blood pressure, as shown in Fig. 4c, d with orange data points. The LOA is [−9.77, 8.14] mmHg for SBP, and for DBP, the LOA is [−9.94, 8.00] mmHg. The SBP measurements show better performance than the DBP measurements. This occurs because of the differences in peripheral resistance of individuals, resulting in differences in pulse waveforms across subjects. Even for the same subject, changes in peripheral resistance due to factors such as exercise and temperature changes cause the pulse waveform to vary over time. In addition, the interface conditions may induce noise in the pulse waveform. These noises and interferences in the pulse waveform cannot be completely eliminated by fusing multichannel sensing signals. In other words, the waveform-based estimation of DBP is more susceptible to environmental interference and results in less robust estimation of DBP than SBP.

As mentioned, pulse waveform analysis (PWA) based on machine learning is a popular method for estimating blood pressure^34,35^. To demonstrate the superiority of our method, we compare the results of our blood pressure estimates with those using the PWA method. The machine learning network of the PWA is composed of a convolutional neural network (CNN), gated recurrent unit (GRU), and dense layers (the details are shown in Supplementary Note S11). The dorsal PPG waveform and the subject’s physical characteristics are the inputs of the PWA network, and the output is SBP or DBP. The estimation error of the PWA method with LOSO reaches −0.61 ± 7.16 mmHg for SBP and −0.57 ± 7.42 mmHg for DBP, as shown in Fig. 4e, f. The blood pressure estimates by the PWA are more scattered than those of our method. It is worth noting that our proposed method has lower computational complexity than the PWA method because we use the extracted features as the network inputs rather than the raw pulse waveform. A lower computational load is more favorable for wearable devices.

To further compare our method with competing approaches for wearable continuous blood pressure measurements, we list numerous state-of-the-art methods^10,14,35–47^ in Supplementary Table S1. Some of these methods^10,35^ use ECG and PPG, which are not convenient for personal use. Most methods evaluate the blood pressure measurement accuracy through subject-specific calibration, and the generalizability of the models has not been evaluated. The above comparisons show that our method achieves better accuracy with good generalizability across subjects for wearable continuous blood pressure monitoring, such that it is superior to the previously demonstrated state-of-the-art methods.

## Conclusion

We present a new method of wearable continuous blood pressure measurement and develop a wearable device that is convenient to wear (like a wristwatch). Utilizing two PPG sensors on the palmar and dorsal sides of the wrist and removing the effect of personal factors by differential operation to detect the cardiac output and combining the interface sensors to detect the wearing contact pressure and skin temperature, continuous blood pressure can be estimated using a simple double-hidden-layer neural network. The model generalizability across individuals has been validated, and the estimation error with LOSO is 0.44 ± 6.00 mmHg for SBP and -0.50 ± 6.20 mmHg for DBP. Compared with other methods, our method achieves better measurement accuracy with good generalization ability. Benefiting from simple wristwatch design and low computational load, our wearable device provides an advantageous solution for continuous BP measurement and greatly enhances the potential for an early finding of patient deterioration. In our future work, we will further improve the following aspects of this wearable device. In terms of the sensor system, we will further optimize its structural design to improve conformity with skin and eliminate environmental interference with PPG sensors. In terms of the algorithm performance, we will further investigate the data-fusion algorithm with optimally extracted features to improve the estimation accuracy for DBP. In addition, the application scenarios considering human exercise and sweating will be further studied in our future work.

Experiments performed in this study involving human participants were approved by the Institution Review Board of Tsinghua University (No. 20180009). Informed consent was obtained from human subjects to use their images and conduct the experiments described in this paper.

### Supplementary information

Revised Supplementary Materials

## Data Availability

The data that support the findings of this study are available from the corresponding author upon reasonable request.
