# Supplementary material for: A new method of continuous blood pressure monitoring using multichannel sensing signals on the wrist
Source: Microsyst Nanoeng. 2023 Sep 21;9:117. doi: 10.1038/s41378-023-00590-4 (PMC10511443; doi:10.1038/s41378-023-00590-4)
Supplement: Supplementary file 1 — Revised Supplementary Materials [file 41378_2023_590_MOESM1_ESM.docx]

Supplementary Materials for

**A new method of continuous blood pressure monitoring using multichannel sensing signals on the wrist**

Liangqi Wang#, Shuo Tian#, Rong Zhu*

State Key Laboratory of Precision Measurement Technology and Instrument, Department of Precision Instrument, Tsinghua University, Beijing, 100084, China.

#The authors contributed equally to this work.

*Corresponding author: Rong Zhu

Email: zr_gloria@mail.tsinghua.edu.cn

#### Note S1. Fabrication of the interface sensor

Cu electrodes and connecting wires of the interface sensor are printed on a flexible polyimide (PI) substrate (DuPont Pyralux AP8525R) by the standard flexible printed circuit technique. Then, the PI substrate is sprayed with photoresist (30 μm, RDMICRO, KXN5735-LO) and patterned by photolithography, a chrome (Cr) film (30 nm) and a platinum (Pt) film (120 nm) are deposited by magnetron sputtering and patterned by a lift-off process. The pattern line width is 80 μm. The Cr layer is used as an adhesion layer to ensure good adhesion between Pt film and polyimide substrate, which improves the mechanical property of the sensor. Finally, the interface sensor is annealed at 200 °C for 2 h in a vacuum oven, and a parylene layer is coated on the sensor by chemical vapor deposition (PDS 2010 Labcoter 2) to make it waterproof and dustproof. The fabrication of the interface sensor is shown in the figure below.


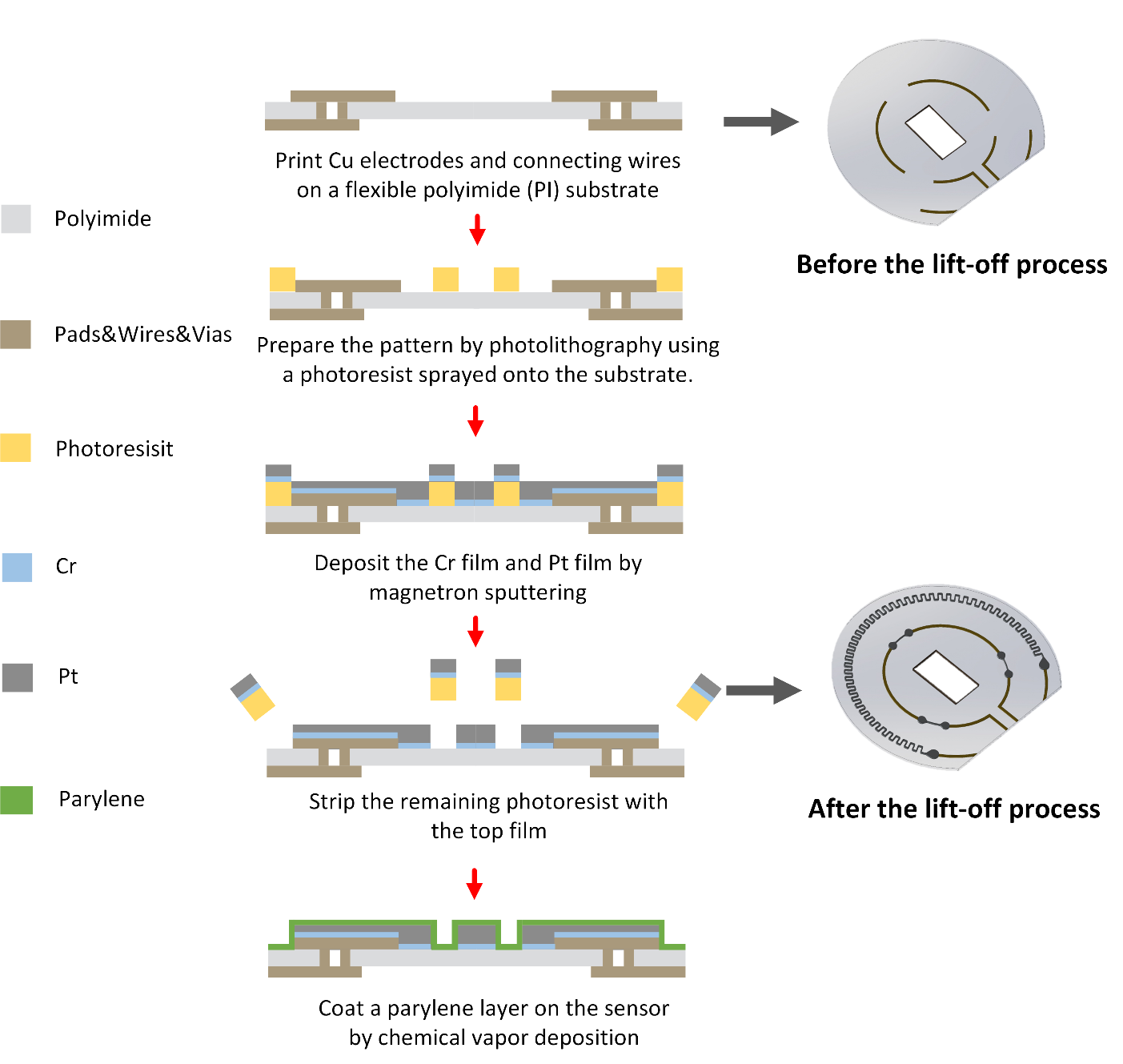


#### Note S2. The principle of temperature compensation by the CTD scheme

Reasonable resistor configuration of the Wheatstone bridge of the CTD circuit allows temperature compensation and decoupling of the bimodal measurements self-sustained for the pressure/temperature or proximity/temperature. As shown in Supplementary Fig.1, when the Wheatstone bridge is balanced, the relationship of resistors of the Wheatstone bridge can be obtained as following:

|  | $R_{\text{b}}\times R_{\text{h}}=R_{\text{a}}\times\left(R_{\text{c}}+R_{\text{ad}}\right)$ | (1-1) |
| --- | --- | --- |

Considering temperature coefficient resistance (TCR) of hot-film (the center ribbon of the interface sensor) and cold-film (the circumjacent ribbon), Equation (1-1) can be expressed as follows:

|  | $R_{\text{b}}\times\left[R_{\text{h0}}\left(1\text{+}\alpha_{\text{h}}T+\alpha_{\text{h}}\Delta T\right)\right]=R_{\text{a}}\times\left[R_{\text{c0}}\left(1\text{+}\alpha_{\text{c}}T\right)+R_{\text{ad}}\right]$ | (1-2) |
| --- | --- | --- |

where $\alpha_{\text{h}}$ and $\alpha_{\text{c}}$ are the TCRs of hot-film and cold-film, and the two are approximately equal (Fig. S2). $R_{\text{h0}}$ and $R_{\text{c0}}$ are the resistances of hot-film and cold-film at $0\text{ }^{\text{o}}\text{C}$, *T* is the ambient temperature, $\Delta T$ is the temperature difference between the hot-film and the environment. Set the resistance ratio of $R_{\text{a}}$ and $R_{\text{b}}$ as:

|  |  | (1-3) |
| --- | --- | --- |

The resistance configuration of the CTD circuit can be derived from Equations (1-1), (1-2), and (1-3):

|  | $\left\{\begin{matrix} \frac{R_{\text{a}}}{R_{\text{b}}}\text{=}\frac{R_{\text{h0}}}{R_{\text{c0}}} \\ \Delta T=\frac{R_{\text{ad}}}{R_{\text{c0}}\alpha_{\text{c}}} \end{matrix}\right.$ | (1-4) |
| --- | --- | --- |

Equation (1-4) indicates temperature difference is independent of the ambient temperature. So the pressure/proximity sensibility is constant and the output voltage *U* is independent of the ambient temperature. $\Delta T$ is set as $5\text{ }^{\text{o}}\text{C}$ in this work.

#### Note S3. The experiment of contact pressure sensing and compression depth sensing

To characterize the pressure sensing capability, the interface sensor is attached to the pressure probe of a force gauge (Sundoo SH-5, 0.01N resolution), and a human wrist is placed on the base of the force gauge. Contact pressure is applied by using a mechanized z-axis stage (Handpi HLD) with the force gauge. The compression depth is controlled by the mechanized z-axis stage. When the pressure probe of the force gauge is in contact with the wrist skin, the compression depth is detected by the interface sensor and the sensor output is recorded.

#### Note S4. The principle of temperature measurement

In our sensor design, a pair of hot-film and cold film is utilized to implement constant temperature difference (CTD) mode by the aid of a CTD circuit. The hot film and cold film are connected into the opposite legs of a Wheatstone bridge. Since the resistance of the cold-film is much larger than that of the hot-film, the Joule heat of the cold-film is negligible according to the CTD circuit. Therefore, the temperature of the cold film is approximately equal to the ambient temperature, and its resistance that related to the ambient temperature can be expressed as following:

|  | $R_{\text{c}}=R_{\text{c0}}\left(1+\alpha_{\text{c}}T\right)$ | (2-1) |
| --- | --- | --- |

where $\alpha_{\text{c}}$ is the TCR of cold-film, $R_{\text{c0}}$is the resistances of cold-film at $0\text{ }^{\text{o}}\text{C}$, *T* is the ambient temperature. According to the circuit shown in Supplementary Fig. 4, the voltage ratio of cold-film can be calculated by:

|  | $\frac{U_{1}}{U_{\text{out}}}=\frac{R_{\text{c}}+R_{\text{ad}}}{R_{\text{c}}+R_{\text{ad}}+R_{\text{b}}}$ | (2-2) |
| --- | --- | --- |

Combining Equation (2-1) and (2-2), the ambient temperature *T* can be expressed as:

|  | $\text{Output\_T=}\frac{U_{1}}{U_{\text{out}}\text{-}U_{1}}=\frac{R_{\text{c0}}}{R_{\text{b}}}\alpha_{\text{c}}T+\frac{R_{\text{ad}}+R_{\text{c0}}}{R_{\text{b}}}$ | (2-3) |
| --- | --- | --- |

#### Note S5. The detailed process of PPG signal processing

First, we remove the high-frequency noise of the PPG signal. A window = 23 and polynomial order = 3 Savitzky-Golay filter is applied to the raw PPG signal, then a clear PPG waveform is obtained by wavelet denoising. Second, a window = 1501 and polynomial order = 2 Savitzky-Golay filter is applied, and the baseline is obtained. We can get the AC component of the PPG signal by subtracting the baseline from the clear PPG waveform. Finally, z-score standardization is performed to transform their mean value to 0 and standard deviation to 1. Not only the PPG signal but also all the features are standardized before inputting to the neural network.

#### Note S6. Definition of the features extracted from PPG signal

The 8 features extracted from PPG signals are defined as follows. The difference in baselines of the two PPG signals is calculated by subtracting the mean value of the baseline of the palmar signal from the dorsal signal. Cardiac period begins with contraction of the atria and ends with ventricular relaxation. In the PPG waveform, the time between the two valleys is the cardiac period. Systolic time (ST) is the time between the left valley and the peak, and diastolic time (DT) is the time between the peak and the right valley. The ratio of systolic area and diastolic area is an indication of vascular tone changes and is related directly to blood pressure(El-Hajj and Kyriacou 2021). K value in a cardiac cycle is calculated by (Pm-Pd)/(Ps-Pd), Pm is the mean value of the PPG waveform, Pd is the mean value of two valleys, and Ps is the peak value. The bigger the K value is, the smoother the waveform is. The ratio of AC and DC components is defined as the ratio of the difference between the peak and valley value and the mean value of the baseline.

#### Note S7. The optimization process of the MLP neural network

In the optimization process of the MLP neural network, PPG baseline difference, contact pressure signals, and the subject’s physical characteristics are used to estimate SBP with LOSO.

In neural network training, it is often found that the error of the model on the training set is much smaller than that on the validation set, and overfitting may occur. To avoid overfitting, a penalty term (L2 regularization) is added to the loss function to perform weight attenuation and limit the sum of squares of the neuron weights. Dropout is used after the hidden layer, and a fixed proportion of neurons will be randomly discarded during each training to make the network insensitive to the weight change of a certain neuron, increase the generalization ability, and reduce overfitting. If the error of the validation set does not decrease after several rounds of training, the training is stopped in time to save the time cost of training, and the trained model is used as the model of the sensor.

The number of layers, the number of neurons, and Dropout of the hidden layer of the neural network are optimized, and the results are shown in the table below. When the number of hidden layers is 1, with the increase of the number of neurons, the absolute value of the average error of systolic blood pressure and the standard deviation of the error are reduced, but the change is not large. With the increase of the number of neurons, the average error and standard deviation gradually decrease and tend to be stable. Therefore, the best MLP structure is a double hidden layer structure, the first hidden layer is 80 neurons, and the second hidden layer is 12 neurons. A Dropout of 20% is applied to each hidden layer, and this neural network will be used later to estimate systolic and diastolic blood pressure.

| neural network structure^*^ | error (ME±SD) | neural network structure | error (ME±SD) |
| --- | --- | --- | --- |
| 15 | -0.67 ± 7.72 | 20(0.2) + 6(0.2) | 0.27 ± 7.16 |
| 20 | -0.48 ± 7.12 | 30(0.2) + 6(0.2) | 0.33 ± 6.93 |
| 20(0.2) | 0.80 ± 7.67 | 30(0.2) + 12(0.2) | 0.38 ± 6.69 |
| 25 | -0.04 ± 7.32 | 30(0.2) + 20(0.2) | 1.01 ± 7.29 |
| 25(0.2) | -0.19 ± 7.24 | 50(0.2) + 12(0.2) | 0.56 ± 6.88 |
| 15+6 | 0.63 ± 7.48 | 80(0.2) + 12(0.2) | 0.24 ± 6.61 |
| 15(0.2) + 6(0.2) | 0.90 ± 7.21 | 80(0.3) + 12(0.2) | 0.20 ± 6.77 |
| 20+6 | 0.27 ± 7.75 |  |  |

*：The number is the number of neurons in the hidden layer, and the number in parentheses is the Dropout ratio

#### Note S8. Details of blood pressure measurement

There are a total of 18 subjects, 13 males and 5 females. The measurement time of each subject is about 1 minute each time, and the measurement interval is at least 3 minutes. Each person measures 10 to 20 times, and the measurement is divided into at least 2 days. The subjects remove and re-wear the monitoring device between different measurements to validate the feasibility and generalization across different wears. There are 309 measurements in total. When measuring, the subject wears the device on the left wrist and wears a commercial cuff-based blood pressure monitor (OMRON J751) on the upper arm of the right hand for simultaneous measurement. The blood pressure measured by the cuff-based blood pressure monitor is used as a ground truth blood pressure.

#### Note S9. Performance metrics

Mean error (ME) refers to the average of all the errors in a set. The error here is the difference between the estimated value and the reference value. It’s defined as:

$$ME=\frac{1}{N}\sum_{i=1}^{N} \left[BP_{\text{est}}\left(i\right)-BP_{\text{ref}}\left(i\right)\right]$$

where *BP*_ref_(*i*) and *BP*_est_(*i*) represent the reference blood pressure and estimated blood pressure in the *i*th time window, and *N* is the number of total time windows.

Standard deviation (SD) is a measure of the amount of variation or dispersion of a set of values. The SD of the measurement error is defined as:

$$SD=\sqrt{\frac{1}{N}\sum_{i=1}^{N} \left(BP_{\text{est}}\left(i\right)-BP_{\text{ref}}\left(i\right)-ME\right)^{2}}$$

Pearson correlation coefficient is a measure of linear correlation between two sets of data. It is the ratio between the covariance of two variables and the product of their standard deviations. It’s defined as:

$$r=\frac{\sum_{i=1}^{N} \left(x_{i}-\overline{x}\right)\left(y-\overline{y}\right)}{\left(N-1\right)s_{x}s_{y}}$$

where *N* is the sample size, *x_i_* and *y_i_* are the individual sample points indexed with *i*, and $\overline{x}$, $\overline{y}$ are the sample mean of each sample.

#### Note S10. Blood pressure estimate results using different parameters

The 5 categories of total 18 features, including dual PPG baseline difference, contact pressure signals and skin temperature signals (palmar and dorsal), subject’s physical characteristics (heart rate, age, height, weight, BMI index, and gender), PPG waveform features set (8 features) are used to estimate SBP and DBP. In the study, 9 more PPG waveform features (systolic width at 25%/50%/75% (SW25, SW50, and SW75), the sum of systolic width and diastolic width at 25%/50%/75% (SW25+DW25, SW50+DW50, and SW75+DW75), the ratio of systolic width and diastolic width at 25%/50%/75% (SW25/DW25, SW50/DW50, and SW75/DW75)) are also used as candidate features to estimate SBP and DBP. It’s named PPG waveform features set B.

Systolic width at 25%/50%/75% (SW25, SW50, and SW75), the sum of systolic width and diastolic width at 25%/50%/75% (SW25+DW25, SW50+DW50, and SW75+DW75), the ratio of systolic width and diastolic width at 25%/50%/75% (SW25/DW25, SW50/DW50, and SW75/DW75), these 9 features are calculated by pulse widths at different amplitudes in systole or diastole, which are correlated with the systemic vascular resistance.^1^

Different combinations of these feature categories are used to estimate blood pressure, the results with LOSO are shown in the table below. The mean absolute error (MAE) and the standard deviation of absolute error (SAE) are also given to evaluate the blood pressure estimates.

| Combinations of feature categories | Estimation error of SBP (mmHg) | Estimation error of DBP (mmHg) |
| --- | --- | --- |
| PPG baseline difference + contact pressure + skin temperature + physical characteristics | 0.44+6.00 (ME±SD) | -0.04±7.09 **(**ME±SD**)** |
|  | 4.89±3.51 **(**MAE±SAE**)** | 5.84±4.02 **(**MAE±SAE**)** |
| PPG baseline difference + contact pressure + skin temperature + physical characteristics + PPG waveform features set | -0.26±7.58 **(**ME±SD**)** | -0.50±6.20 (ME±SD**)** |
|  | 6.21±4.36 **(**MAE±SAE**)** | 5.12±3.52 **(**MAE±SAE**)** |
| PPG baseline difference + contact pressure + skin temperature + physical characteristics + PPG waveform features set B | 0.91±7.05 **(**ME±SD**)** | -0.81±6.26 **(**ME±SD**)** |
|  | 5.63±4.34 **(**MAE±SAE**)** | 5.38±3.81 **(**MAE±SAE**)** |

It can be seen from the above results that for SBP, the combination of "PPG baseline difference + contact pressure + skin temperature + physical characteristics" has the lowest error. For DBP, the lowest error is estimated using the combination of "PPG baseline difference + contact pressure + skin temperature + physical characteristics + PPG waveform features set".

#### Note S11. Details of deep learning neural network for PWA

To validate the advantage of our proposed method, we compare the result of blood pressure estimates by using our proposed method with the result using the PWA method. The deep learning network framework of the PWA refers to the framework proposed in the related literature^2,3^, and is optimized to use a deep learning network combining convolutional neural network (CNN), Gated recurrent unit (GRU) and Dense with our dataset. It is shown in the figure below.


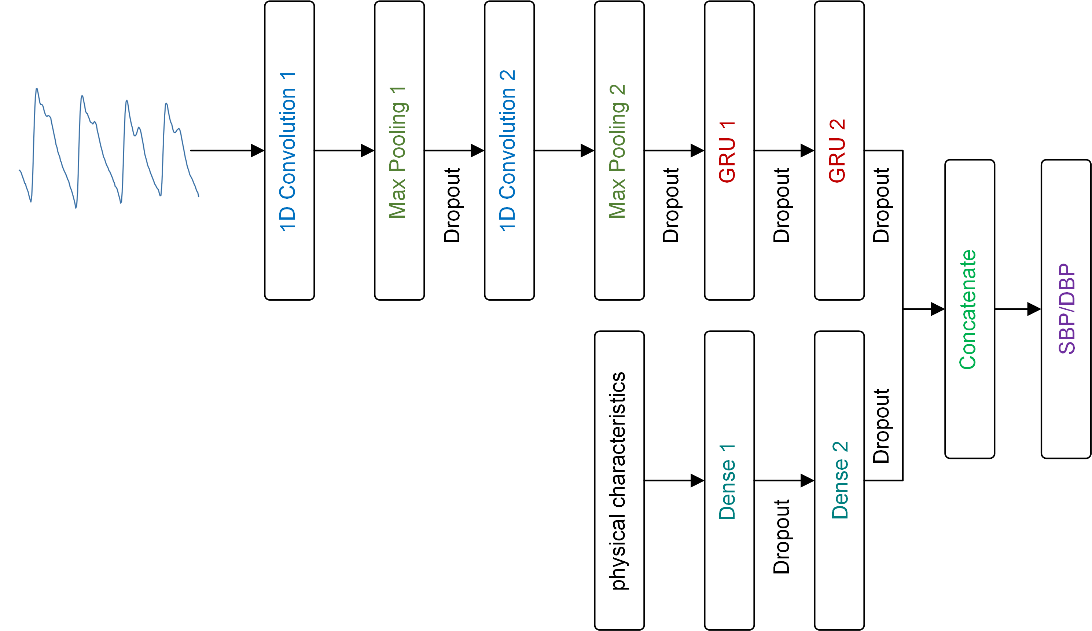


The input of this network is PPG waveform (dorsal PPG sensor signal) and subject physical characteristics (heart rate, height and weight, etc.), and the output is SBP or DBP. The one-dimensional convolutional layer is used to process the PPG waveform and extract features, and the two convolutional layers each have 20 convolution kernels with a size of 9. The pooling layer is used to preserve the main features while reducing the number of parameters and calculations to prevent overfitting. Here, the commonly used max pooling with size 5 is used. The pooling layer is followed by two layers of GRUs, each with 10 units, for further feature extraction and regression operation. Each pooling layer is followed by 50% Dropout and each GRU layer is followed by 40% Dropout. The fully connected layers Dense 1 and Dense 2 that connect the feature signal input layer use the feature signal for regression operation, the number of neurons in Dense 1 layer is 80, and the number of neurons in Dense 2 layer is 12. Both fully connected layers are followed by 20% Dropout. Finally, the output of GRU 2 is concatenated with Dense 2, and finally the systolic or diastolic blood pressure is output. All hyperparameters in the network have been optimized.

#### Fig. S1. Details of the resistances of the interface sensor. The temperature coefficient of resistance (TCR) of a thermistor is defined as $\alpha=\Delta R/\left(R_{0}\times\Delta T\right)$, where $R_{0}$ is the resistance at $0\text{ }^{\text{o}}\text{C}$, $\Delta R$ refers to the resistance change when the temperature change is $\Delta T$. The TCRs of $R_{\text{c}}$ and $R_{\text{h}}$ of interface sensor 1 are $2.17\times10^{-3}\text{K}^{\text{-1}}$ and $2.07\times10^{-3}\text{K}^{\text{-1}}$, respectively. The TCRs of $R_{\text{c}}$ and $R_{\text{h}}$ of interface sensor 2 are $2.16\times10^{-3}\text{K}^{\text{-1}}$ and $2.18\times10^{-3}\text{K}^{\text{-1}}$, respectively.


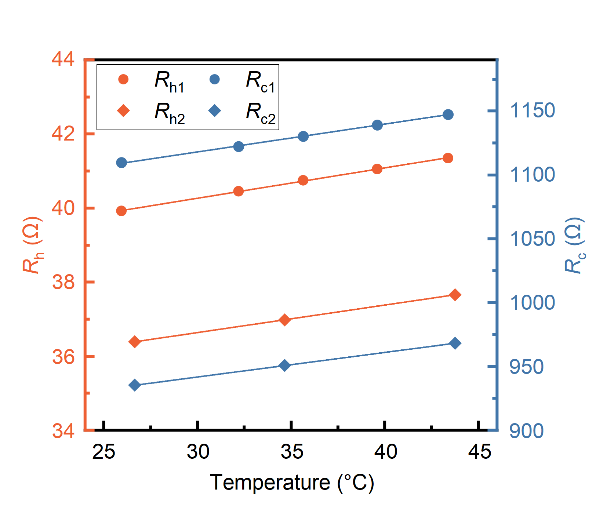


**Fig. S2. Schematic of the CTD circuit.** Two Pt ribbons are connected into two legs of a Wheatstone bridge. $R_{\text{h}}$denotes the hot film and $R_{\text{c}}$ denotes the cold film. $R_{\text{a}}$ and $R_{\text{b}}$ are two fixed resistors. $R_{\text{ad}}$ is an adjustable resistor for adjusting the sensitivity of the interface sensor. $U$ denotes the top voltage of the bridge. $U_{1}$ and $U_{2}$ denote the input voltages of an instrumentation amplifier.


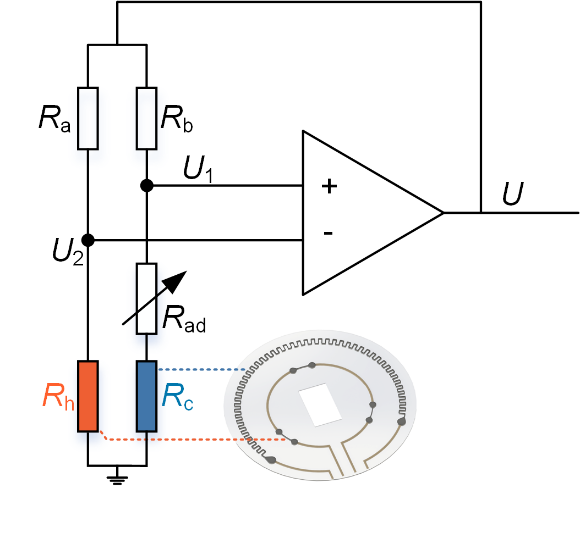


#### Fig. S3. Testing results of temperature compensation for the pressure sensing of the interface sensor. Temperature effect on proximity/pressure sensing response is less than 4 mV for the interface sensor 1 from $27\text{ }^{\text{o}}\text{C}$ to $45\text{ }^{\text{o}}\text{C}$. Temperature effect on proximity/pressure sensing response is less than 10 mV for the interface sensor 2 from $27\text{ }^{\text{o}}\text{C}$ to $45\text{ }^{\text{o}}\text{C}$. The pressure errors of the interface sensors induced from the temperature variation are less than 2%.


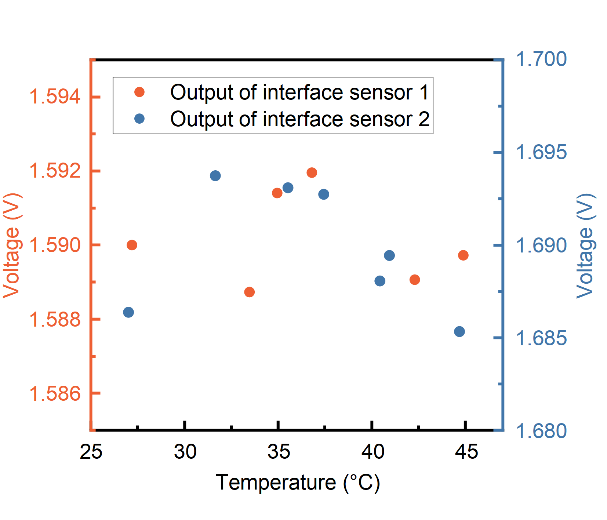


#### Fig. S4. Temperature sensing of the interface sensor. Temperature output of the interface sensors from $27\text{ }^{\text{o}}\text{C}$ to $45\text{ }^{\text{o}}\text{C}$. They have a linear relationship with temperature.


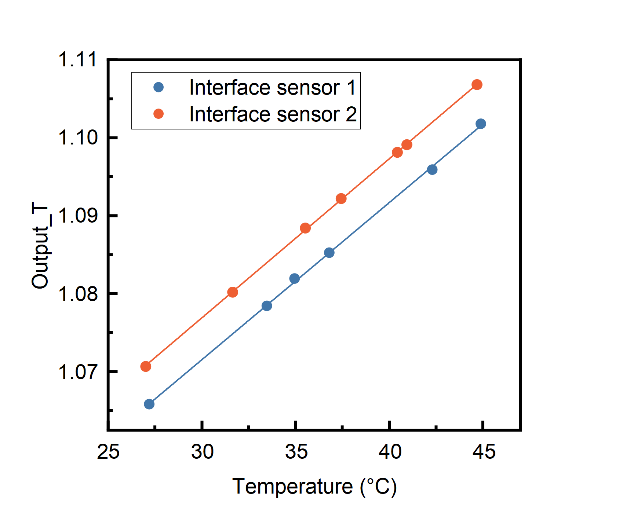


#### Fig. S5. Photograph of blood pressure measurement. The wristwatch-like measuring device was used to measure the blood pressure of the subjects. The device was worn on the left wrist for measurement, and the commercial cuff sphygmomanometer (Omron J751) was worn on the right upper arm for simultaneous measurement. The blood pressure shown on the cuff sphygmomanometer was used as the actual blood pressure for reference.


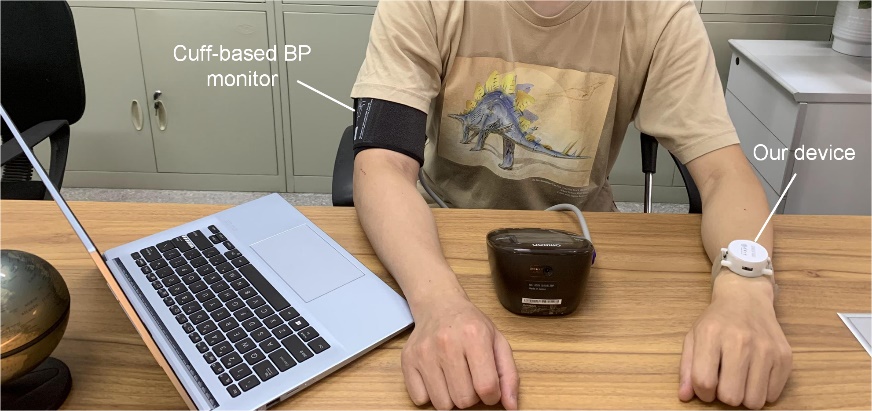


#### Fig. S6. The blood pressure distribution. The systolic blood pressure mainly ranges from 90 to 130 mmHg, and the diastolic blood pressure mainly ranges from 55 to 80 mmHg.

#### Table S1. The reported methods of wearable continuous blood pressure measurement

| BP estimation method | Data source – Number of subjects | Sensor type  - Portability | Generalization | SBP estimation error (mmHg) | DBP estimation error (mmHg) | Ref |
| --- | --- | --- | --- | --- | --- | --- |
| Wrist dual PPG combined with interface pressure and temperature | Own experiments - 18 | Wristwatch (dual PPG + interface sensors)  - High | Generalization across individuals**  (With LOSO) | 0.44±6.00 (ME+SD)* | -0.50±6.20 (ME+SD) | This work |
|  |  |  |  | 4.89±3.51 (MAE±SAE)* | 5.12±3.52 (MAE±SAE) |  |
| Wrist dual PPG combined with interface pressure and temperature | Own experiments - 18 | Wristwatch (dual PPG + interface sensors)  - High | Model trained and tested by different datasets of the same subject group (Without LOSO ) | -0.81±4.57 (ME+SD) | -0.97±4.58 (ME+SD) | This work |
|  |  |  |  | 3.75±2.73 (MAE±SAE) | 3.55±3.05 (ME+SD) |  |
| PWV | Own experiments - 35 | Single device at carotid artery  - High | Generalization across individuals | 1.15±7.98 (ME+SD) | 0.86±6.36 (ME+SD) | 4 |
| PWA | MIMIC-II database - 45 | Non-portable multi-lead ECG + PPG finger clip -Low | Generalization across individuals | 4.43±6.09 (MAE±SAE) | 3.32±4.75 (MAE±SAE) | 3 |
| PWA | MIMIC-II database - 942 | Non-portable multi-lead ECG + PPG finger clip -Low | Generalization across individuals | 9.30±8.85 (MAE±SAE) | 5.12±5.52 (MAE±SAE) | 5 |
| PWA | MIMIC-III database - 510 | PPG finger clip  -High | Generalization across individuals | 9.43 (MAE) | 6.88 (MAE) | 6 |
| PAT+ PWA | MIMIC-II database - 942 | Non-portable multi-lead ECG + PPG finger clip -Low | Generalization across individuals | 11.8±9.87 (MAE±SAE) | 5.83±5.71 (MAE±SAE) | 7 |
| PTT+  PWA | Own experiments - 27 | Single lead ECG on the left and right arms + PPG finger clip  – Low | Subject-specific model*** | -0.37±5.21 (ME±SD) | -0.08±4.06 (ME±SD) | 8 |
| PAT+ PWA | Own experiments - 85 | Non-portable multi-lead ECG + wrist and ankle PPG -Low | Subject-specific model | 1.62±7.76 (ME±SD) | 1.49±5.52 (ME±SD) | 9 |
| PTT+ PWA | Own experiments - 7 | Wrist multi-pair electrical impedance sensor - Low | Subject-specific model | 0.17±5.77 (ME±SD) | 0.16±4.47 (ME±SD) | 10 |
| PWA | MIMIC-II/III database - 15 | PPG finger clip -High | Subject-specific model | -0.00±6.00 (ME±SD) | 0.00±3.30 (ME±SD) | 11 |
| PTT | Own experiments - 20 | Fingertip multi-wavelength PPG - High | Subject-specific model | 1.86±2.85 (MAE±SAE) | 1.49±1.75 (MAE±SAE) | 12 |
| PTT+ PWA | Own experiments - 73 | Arm and leg single lead ECG + PPG finger clip - Low | Subject-specific model | 0.00±3.10 (ME±SD) | 0.00±2.20 (ME±SD) | 13 |
| PWA | MIMIC-II database - 1157 | PPG finger clip  -High | Not stated | 1.55±5.41 (ME±SD) | -1.25±5.65 (ME±SD) | 2 |
| PTT+ PWA | MIMIC-I - database - 39 | Non-portable multi-lead ECG + PPG finger clip -Low | Model trained and tested by different datasets of the same subject group | 0.02±1.56 (ME±SD) | 0.01±0.85 (ME±SD) | 14 |
| PWA | Own experiments - 85 | Wrist piezoresistive pressure sensor - Low | Not stated | 0.00±3.06 (ME±SD) | 0.10±2.77 (ME±SD) | 15 |
| PWA | Own experiments - 7 | Wrist capacitive pressure sensor - Low | Subject-specific model | -0.05±2.09 (ME±SD) | | 16 |
| PWA | Own experiments - 3 | Neck ultrasound sensor patch  - Low | Subject-specific model | -2.0 (ME) | -0.3 (ME) | 17 |

*ME is mean error. SD is standard deviation. MAE is mean absolute error. SAE is standard deviation of absolute error.

** Generalization across individuals refers to the generalized model is trained and tested by different subjects.

*** Subject-specific model refers to the individual model is trained and tested by the same subject.

References

1. El-Hajj, C. & Kyriacou, P. A. Deep learning models for cuffless blood pressure monitoring from PPG signals using attention mechanism. *BIOMEDICAL SIGNAL PROCESSING AND CONTROL* **65,** 102301; 10.1016/j.bspc.2020.102301 (2021).

2. Panwar, M., Gautam, A., Biswas, D. & Acharyya, A. PP-Net: A Deep Learning Framework for PPG-Based Blood Pressure and Heart Rate Estimation. *IEEE Sensors Journal* **20,** 10000–10011; 10.1109/JSEN.2020.2990864 (2020).

3. Yang, S., Zhang, Y., Cho, S.-Y., Correia, R. & Morgan, S. P. Non-invasive cuff-less blood pressure estimation using a hybrid deep learning model. *Optical and Quantum Electronics* **53,** 93; 10.1007/s11082-020-02667-0 (2021).

4. Nabeel, P. M., Jayaraj, J. & Mohanasankar, S. Single-source PPG-based local pulse wave velocity measurement: a potential cuffless blood pressure estimation technique. *Physiological measurement* **38,** 2122–2140; 10.1088/1361-6579/aa9550 (2017).

5. Baek, S., Jang, J. & Yoon, S. End-to-End Blood Pressure Prediction via Fully Convolutional Networks. *IEEE Access* **7,** 185458–185468; 10.1109/ACCESS.2019.2960844 (2019).

6. Slapničar, G., Mlakar, N. & Luštrek, M. Blood Pressure Estimation from Photoplethysmogram Using a Spectro-Temporal Deep Neural Network. *Sensors* **19,** 3420; 10.3390/s19153420 (2019).

7. Mohammad, K., Mohammad, M. K., Hoda, M. & Mahdi, S. Cuffless Blood Pressure Estimation Algorithms for Continuous Health-Care Monitoring. *IEEE Transactions on Biomedical Engineering* **64,** 859–869; 10.1109/TBME.2016.2580904 (2017).

8. Ding, X.-R., Zhang, Y.-T., Liu, J., Dai, W.-X. & Tsang, H. K. Continuous Cuffless Blood Pressure Estimation Using Pulse Transit Time and Photoplethysmogram Intensity Ratio. *IEEE Transactions on Biomedical Engineering* **63,** 964–972; 10.1109/TBME.2015.2480679 (2016).

9. Fen, M., Zeng-Ding, L., Ji-Kui, L. & Bo, W. Multi-Sensor Fusion Approach for Cuff-Less Blood Pressure Measurement. *IEEE journal of biomedical and health informatics* **24,** 79–91; 10.1109/JBHI.2019.2901724 (2020).

10. Kireev, D. *et al.* Continuous cuffless monitoring of arterial blood pressure via graphene bioimpedance tattoos. *Nature Nanotechnology* **17,** 864–870; 10.1038/s41565-022-01145-w (2022).

11. Lin, W.-H. *et al.* Towards accurate estimation of cuffless and continuous blood pressure using multi-order derivative and multivariate photoplethysmogram features. *BIOMEDICAL SIGNAL PROCESSING AND CONTROL* **63,** 102198; 10.1016/j.bspc.2020.102198 (2021).

12. Liu, J. *et al.* Multi-Wavelength Photoplethysmography Enabling Continuous Blood Pressure Measurement With Compact Wearable Electronics. *IEEE Transactions on Biomedical Engineering* **66,** 1514–1525; 10.1109/TBME.2018.2874957 (2019).

13. Miao, F. *et al.* A Novel Continuous Blood Pressure Estimation Approach Based on Data Mining Techniques. *IEEE journal of biomedical and health informatics* **21,** 1730–1740; 10.1109/JBHI.2017.2691715 (2017).

14. Tanveer, M. S. & Hasan, M. K. Cuffless blood pressure estimation from electrocardiogram and photoplethysmogram using waveform based ANN-LSTM network. *BIOMEDICAL SIGNAL PROCESSING AND CONTROL* **51,** 382–392; 10.1016/j.bspc.2019.02.028 (2019).

15. Zhang, Q. *et al.* Highly sensitive resistance-type flexible pressure sensor for cuffless blood-pressure monitoring by using neural network techniques. *Composites Part B: Engineering* **226,** 109365; 10.1016/j.compositesb.2021.109365 (2021).

16. Kim, J. *et al.* Soft Wearable Pressure Sensors for Beat-to-Beat Blood Pressure Monitoring. *Advanced healthcare materials* **8,** e1900109; 10.1002/adhm.201900109 (2019).

17. Wang, C. *et al.* Monitoring of the central blood pressure waveform via a conformal ultrasonic device. *Nature biomedical engineering* **2,** 687–695; 10.1038/s41551-018-0287-x (2018).
